# Ultra-broadband near-field Josephson microwave microscopy

**DOI:** 10.1093/nsr/nwae308

**Published:** 2024-09-03

**Authors:** Ping Zhang, Yang-Yang Lyu, Jingjing Lv, Zihan Wei, Shixian Chen, Chenguang Wang, Hongmei Du, Dingding Li, Zixi Wang, Shoucheng Hou, Runfeng Su, Hancong Sun, Yuan Du, Li Du, Liming Gao, Yong-Lei Wang, Huabing Wang, Peiheng Wu

**Affiliations:** School of Electronic Science and Engineering, Nanjing University, Nanjing 210023, China; School of Electronic Science and Engineering, Nanjing University, Nanjing 210023, China; School of Electronic Science and Engineering, Nanjing University, Nanjing 210023, China; School of Electronic Science and Engineering, Nanjing University, Nanjing 210023, China; Purple Mountain Laboratories, Nanjing 211111, China; School of Electronic Science and Engineering, Nanjing University, Nanjing 210023, China; School of Electronic Science and Engineering, Nanjing University, Nanjing 210023, China; Purple Mountain Laboratories, Nanjing 211111, China; School of Electronic Science and Engineering, Nanjing University, Nanjing 210023, China; School of Electronic Science and Engineering, Nanjing University, Nanjing 210023, China; School of Electronic Science and Engineering, Nanjing University, Nanjing 210023, China; School of Electronic Science and Engineering, Nanjing University, Nanjing 210023, China; School of Electronic Science and Engineering, Nanjing University, Nanjing 210023, China; Purple Mountain Laboratories, Nanjing 211111, China; School of Electronic Science and Engineering, Nanjing University, Nanjing 210023, China; School of Electronic Science and Engineering, Nanjing University, Nanjing 210023, China; School of Materials Science and Engineering, Shanghai Jiao Tong University, Shanghai 200240, China; School of Electronic Science and Engineering, Nanjing University, Nanjing 210023, China; Purple Mountain Laboratories, Nanjing 211111, China; School of Electronic Science and Engineering, Nanjing University, Nanjing 210023, China; Purple Mountain Laboratories, Nanjing 211111, China; School of Electronic Science and Engineering, Nanjing University, Nanjing 210023, China; Purple Mountain Laboratories, Nanjing 211111, China

**Keywords:** Josephson junction, nanoprobe, near-field microwave, frequency mixing

## Abstract

Advanced microwave technologies constitute the foundation of a wide range of modern sciences, including microwave integrated circuits, quantum computing, microwave photonics, spintronics, etc. To facilitate the design of chip-based microwave devices, there is an increasing demand for state-of-the-art microscopic techniques that are capable of characterizing near-field microwave distribution and performance. In this work, we integrate Josephson junctions onto a nanosized quartz tip, forming a highly sensitive microwave mixer on-tip. This allows us to conduct spectroscopic imaging of near-field microwave distributions with high spatial resolution. By leveraging its microwave-sensitive characteristics, our Josephson microscopy achieves a broad detecting bandwidth of ≤200 GHz, as well as remarkable frequency and intensity resolutions. Near-field characterizations of microwave circuits are also conducted to demonstrate the capabilities of Josephson microscopy. Our work emphasizes the benefits of utilizing Josephson microscopy as a real-time, non-destructive technique to advance integrated microwave devices.

## Teaser

Josephson microwave microscopy integrating Josephson junctions onto a nanoprobe enables spectroscopic imaging of near-field microwave with a broad bandwidth, presenting a non-destructive technique to characterize microwave devices.

## INTRODUCTION

Microwave technology, based on precise generation, manipulation and measurement of microwaves, plays a pivotal role across various fields, including microwave integrated circuits [1–4], circuit quantum electrodynamics [5–7], microwave photonics [8,9], spintronics [10,11], etc. These devices are primarily reliant on precise microwave control, of which advancements often depend on the exploration of nanoscale microwave materials and underlying mechanisms. Meanwhile, these integrated devices often demand efficient microwave transmission with supporting planar microcircuits [12], where the integration poses challenges to electromagnetic incompatibility and signal crosstalk. For purposes of achieving optimal performance, there is a growing need to detect near-field microwave distribution from microwave devices, aiming to analyse mechanisms of microwave interaction and facilitate efficient signal coupling.

Presently, several microwave imaging techniques have been developed, including scanning near-field microwave microscopy (SNMM) [13–16], nitrogen-vacancy (NV) center microscopy [17–19], atomic vapor cell microscopy [20,21], etc. Conventional SNMM, for instance, relies on a metal-coated tip to facilitate microwave coupling and transmission along radio frequency (RF) cables. The microwave loss during this process becomes more severe with a decrease in the tip size, as well as an increase in microwave frequency, so it is difficult to balance the sensitivity and spatial resolution when detecting the weak microwave emissions from the device surface. High-sensitivity microscopy, taking NV-center microscopy as the most representative technique, typically requires external magnetic fields to broaden the frequency band, especially when there is a need to measure microwave signals of up to several tens of gigahertz [19,22]. The biasing magnetic fields can substantially degrade magnetic-sensitive devices and their associated circuits. Consequently, there is an urgent requirement for the development of a highly sensitive and field-free microwave microscopic technique.

The Josephson junction refers to a structure in which two superconductors are separated by a thin insulator or a normal conductor, which is an important element in superconducting electronics. It constitutes a weakly coupled junction in which Cooper pairs can tunnel through the barrier via the tunneling effect, traversing from one side to the other [23]. The profoundly non-linear characteristics of Josephson current can be used as mixers [24], with their sensitivity approaching the quantum noise limit [25]. Furthermore, when the characteristic frequency of Josephson junctions is exceeded, it can be used as a bolometer [26] and exhibits exceptional sensitivity to microwaves [27], and is even capable of detecting single microwave photons [28,29]. However, conventional on-chip Josephson junctions cannot be brought in close proximity to devices at the limit, thus making it challenging to achieve near-field imaging with high spatial resolution. Previous works fabricated superconducting quantum interference devices (SQUIDs) onto nanoscale tips to achieve the high-spatial-resolution detection of static magnetic fields [30,31] and thermal phenomena [32,33], providing a new idea for exploring microscopic physical phenomena through the utilization of Josephson junctions.

**Figure 1.**
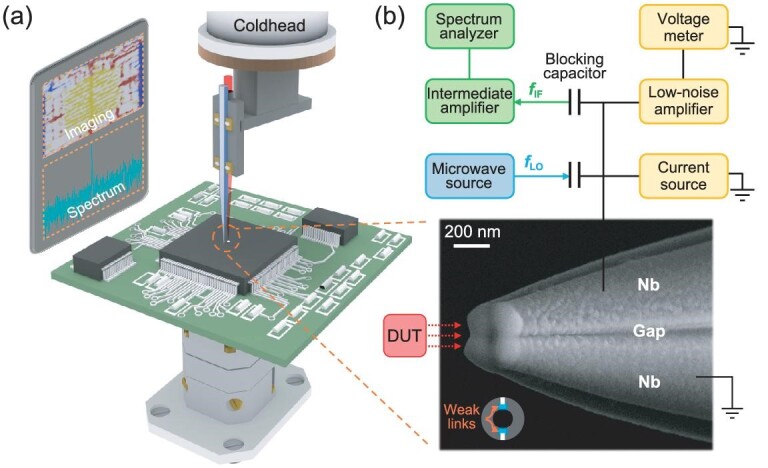
Demonstration of the Josephson microscope. (a) Microscope assembly. When the probe approaches the devices under test (DUT), weak-link Josephson junctions on the apex receive subtle near-field microwaves that are emitted from the DUT surface, causing oscillations of Cooper pairs. The probe is biased by a current source to perform coherent detection and characterizations of microwave distribution and spectrum tracking. (b) Measurement system. The scanning electron microscope (SEM) image of the probe (bottom) shows the formation of weak-link Josephson junctions on top separated by grooves. External measuring circuits are divided into direct current (DC) and alternating current (AC) modules separated by blocking capacitors (top). The DC module employs customized low-noise source meters for probe biasing and readout. AC signals (*f*_IF_ and *f*_LO_) are transmitted through DC-blocking capacitors to conduct frequency mixing when the Josephson probe receives microwaves (*f*_RF_) from the DUT.

Here, we develop an innovative Josephson microscope by integrating Josephson junctions directly onto a nanoprobe, thereby enabling efficient low-temperature near-field microwave characterization (Fig. 1a). The Josephson microscope demonstrates broadband coherent detection with frequencies of ≤200 GHz, as well as a power sensitivity of –72 dBm and a spatial resolution of sub-micrometers. Notably, passive detection ensures non-destructive imaging and *in situ* characterization without external biasing fields, thereby keeping the normal operational state of devices under test (DUT), especially for highly sensitive devices.

## RESULTS

### Microwave sensing

Traditional methods for fabricating probes, such as directional electron beam evaporation [34] or focused ion beam milling [35], are typically used to create nano-SQUIDs. There is also a method that involves a specially designed collimated sputtering system to manufacture MoRe-alloy probes [36]. In our approach, we utilize a quartz tip with deep grooves around the sidewall to produce high-quality Nb probes via commonly available direct current (DC) magnetron sputtering (Fig. S1). Those grooves serve to separate the Nb films, effectively addressing the challenges posed by the isotropic nature of magnetron sputtering, thus forming weak links at the apex (bottom panel of Fig. 1b). Our method facilitates the mass production of Josephson probes. The characterization system of the Josephson microscope consists of DC and alternating current (AC) modules (upper panel of Fig. 1b). Detailed descriptions can be found in the Supplementary Data.

**Figure 2.**
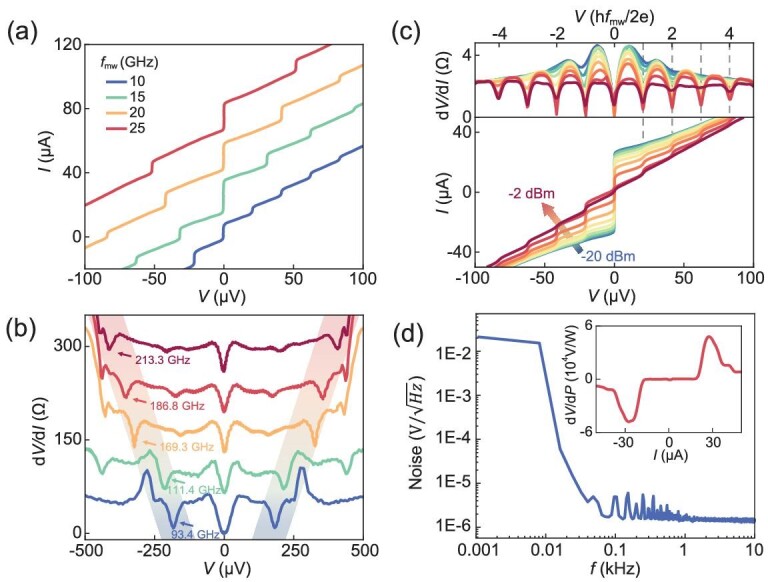
Microwave response of Josephson probes. (a) Microwave response for Probe #1. Shapiro steps at different positions were presented in *I*–*V* characteristics when the probe was exposed to microwaves with frequencies of 10/15/20/25 GHz. The curves were shifted along the *y*-axis (25-μA intervals) for clarity. (b) Microwave response for Probe #2. Shapiro steps were observed as dips in the voltage dependence of differential resistances when being exposed to microwaves of >100 GHz. The features are marked out by arrows with corresponding microwave frequencies. The curves were shifted along the *y*-axis (65-Ω intervals) for clarity. (c) Microwave intensity dependence of *I*–*V* characteristics for Probe #1. The lower graph shows the Shapiro steps when the probe is irradiated by 10 GHz microwaves with an intensity of –20 to –2 dBm. The upper graph shows the voltage dependence of the differential resistance corresponding to the *I*–*V* characteristics below. (d) Noise voltage spectral density for Probe #1. The probe is biased around the critical current *I*_c_ and the white-noise level reaches a minimum value of 1.5 μV Hz^–1^^/2^. The microwave responsivity of the probe that is shown in the inset is obtained by subtracting two *I*–*V* characteristics shown in Fig. S4 and considering the attenuation coefficient. All measurements mentioned above were conducted at a bath temperature of 3.3 K.

The transport performance of the Josephson probe is first demonstrated to evaluate the capability of microwave sensing. Here, we take Probe #1 as a demonstration. The probe has a superconducting critical temperature (*T*_c0_) of ∼5 K and exhibits non-hysteretic current–voltage (*I*–*V*) characteristics (Fig. S2a and b), which is a typical feature of superconducting Dayem-bridges. All measurements were conducted at a bath temperature of 3.3 K. Under microwave irradiations, the Josephson probe demonstrates resonance phenomena. This resonance is characterized by discrete current steps in its *I*–*V* characteristics [37], denoted as Shapiro steps (Fig. 2a). The voltage positions of the Shapiro steps follow the AC Josephson voltage–frequency relationship of 2e/h = 483.6 MHz/μV (where h is the Planck constant and e is the elementary charge), while the width of the Shapiro steps is relevant to the microwave intensity. Shapiro steps indicate the existence of Josephson junctions at the apex of the probe (Fig. S3), confirming the ability of microwave coherent detection at the frequencies of the signal applied. The highest microwave frequency that the Josephson probe can currently detect is >200 GHz. Taking Probe #2 as another example, it also shows non-hysteretic transport characteristics (Fig. S2c and d). Under the irradiation at each frequency, the curves of differential resistance exhibit dips at corresponding voltages (Fig. 2b), representing the Shapiro steps. The frequency range of the detectable microwaves is determined by the product of the superconducting critical currents (*I*_c_) and normal resistance (*R*_n_) of the probe, which will be discussed in the frequency-tracking section.

To accurately describe the intensity resolution, we used a commercial microwave source to measure the *I*–*V* characteristics of Probe #1. Figure 2c shows a clear visualization of the voltage response and differential resistance as a function of the probe voltages and microwave powers. The observed current steps on the *I*–*V* characteristics correspond to the minima of the d*V*/d*I* values, following exactly the positions at integer times of h*f*_mw_/2e, where *f*_mw_ is 10 GHz in this case. As the microwave signal is emitted in space, there exists an enormous loss due to the spatial coupling between the RF cable and the probe. We fit *I*–*V* characteristics by using the classic resistively and capacitively shunted junction model (Fig. S4). The original *I*–*V* characteristic is fitted to obtain a Stewart–McCumber parameter of 0.1, confirming a non-hysteretic feature. After fitting the microwave response of the *I*–*V* characteristic of the probe, the attenuation coefficient of the whole system can be calculated to be 50.6 dB. To evaluate the intensity resolution of the Josephson probe, we obtain the voltage of the probe as it changes with the microwave power, denoted as d*V*/d*P*, with variation in the bias currents (inset of Fig. 2d). The highest microwave response is observed near the critical current of the probe (*I*_c_ ∼ 30 μA), with a maximum value of ≤2.41 × 10^4^ V/W. Then, the noise voltage spectral density is tracked with a bias current around the *I*_c_ of the probe (Fig. 2d). The voltage noise displays a transition from low-frequency 1/*f* noise to high-frequency white noise, with a voltage noise level of ∼1.5 μV Hz^–1^^/2^ in the kHz range. Taking into account the voltage noise and the responsivity of the probe, the sensitivity of the probe is around –72.1 dBm Hz^–1^^/2^ at 3.3 K. Consequently, the Josephson probe demonstrates exceptional adaptability to microwave chips with low power consumption.

### Frequency tracking

**Figure 3.**
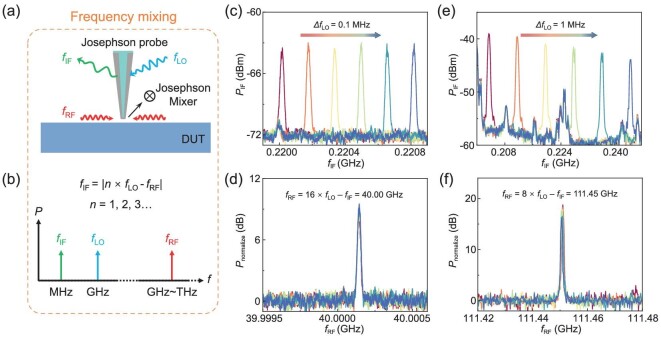
Frequency tracking by the Josephson probe. (a) Principle of the frequency mixing. The Josephson probe acts as a mixer and receives *f*_RF_ signals from the DUT surface. By interacting with a biased local oscillation at *f*_LO_, the probe transforms them into *f*_IF_ outputs. (b) Frequency bands of involved signals. The frequency bands of *f*_IF_ and *f*_LO_ are located in MHz and GHz, respectively, while the *f*_RF_ signal could reach up to GHz or even THz. Three signals satisfy the relationship of *f*_IF_ = |*n* × *f*_LO_ ± *f*_RF_|. (c) Harmonic frequency mixing for Probe #1. The provided *f*_LO_ is changing from 2.51 372 to 2.51 386 GHz with an interval of 0.1 MHz (from left to right). Peaks of the spectra indicate that the corresponding *f*_IF_ outputs shift by an interval of 1.6 MHz, indicating Δ*f*_IF_ = 16*Δ*f*_LO_. (d) Restored spectra of *f*_RF_. By regarding *f*_LO_ as a single frequency output and fitting the equation of *f*_RF_ = 16 × *f*_LO_ – *f*_IF_, the spectra of *f*_RF_ could be exactly reconstructed from (c). (e) Harmonic frequency mixing for Probe #2. Peaks of the spectra indicate that the shift in *f*_IF_ output is 8 MHz in each step, while *f*_LO_ is changing from 13.9568 to 13.9618 GHz with an interval of 1 MHz (from left to right). (f) Restored spectra of *f*_RF_. By fitting the equation of *f*_RF_ = 8 × *f*_LO_ – *f*_IF_, the unknown *f*_RF_ is estimated to be ∼111.45 GHz.

The highly non-linear nature of Josephson junctions makes them exceptional mixers. Unlike conventional Josephson junction mixers that receive signals from a far field, the Josephson probe integrates a nanosized Josephson mixer onto a tip, enabling near-field microwave detection. Specifically, this technique allows frequency tracking towards weak microwave signals based on fundamental or harmonic frequency mixing (Fig. 3a), effectively eliminating the effects of transmission losses and noise interference. When the Josephson probe receives a radio frequency (*f*_RF_) input from the DUT, it generates a frequency mixing product with the local oscillation (*f*_LO_) input provided by a microwave source, typically operating within a range of a few GHz. Subsequently, the resultant intermediate frequency (*f*_IF_) output is amplified and collected by using a spectral analyser. This method provides a means of calculating high-frequency *f*_RF_ (∼GHz/THz) signals by detecting low-frequency *f*_IF_ (∼MHz) signals (Fig. 3b). When the Josephson probe detects both *f*_LO_ and *f*_RF_ signals, its intrinsic nonlinearity generates *f*_IF_ signals given by |*mf*_RF_ ± *nf*_LO_|, where *m* and *n* are positive integers. Here, we focus on the low-frequency output with *m* = 1, resulting in *f*_IF_ = |*nf*_LO_*—f*_RF_|, which is in the range of hundreds of MHz and easier for amplification and detection. By capturing the corresponding *f*_IF_, we can deduce the frequency of the DUT via *f*_RF_ = *nf*_LO_ ± *f*_IF_, where + or – can be easily decided by the change of *f*_IF_ compared with that of *f*_LO_.

Figure 3c presents the capability of the probe for frequency tracking by Josephson mixing. To demonstrate the process, we generate a replica of an ‘unknown’ *f*_RF_ signal using another microwave source. A microwave signal of 40 GHz is involved for Probe #1 (Fig. 3c). For every 0.1-MHz change in the *f*_LO_ signal, the *f*_IF_ output shifts by ∼1.6 MHz, indicating a 16th harmonic mixing. By iteratively adjusting the frequency of *f*_LO_ and collating measurement results, we determined an average value of *f*_RF_ to be precisely 40 GHz ± 2.2 kHz (Fig. 3d). Fundamental frequency mixing is also conducted with *f*_LO_ close to *f*_RF_ and the results present the same frequency value of 40 GHz (Fig. S5). The counts of values are divided into only two columns, showing the limitation of our spectral analyser and the possibility for further optimization. For Probe #2, a microwave signal is generated by using a Gunn oscillator and emitted onto the probe. The frequency of the microwave is ∼112 GHz, yet the accurate value remains to be measured. The *f*_LO_ signals change with an interval of 1 MHz, while the corresponding *f*_IF_ signals vary by 8 MHz in each step (Fig. 3e). By fitting the equation of *f*_RF_ = 8 × *f*_LO_ – *f*_IF_, the unknown frequency is calculated to be ∼111.45 GHz (Fig. 3f). As the amplitude of *f*_LO_ remains unchanged, the amplitude and frequency of the *f*_IF_ output only depend on those of *f*_RF_. Therefore, we can achieve frequency-selective intensity detection based on frequency mixing, e.g. resolving the intensity distribution of certain frequencies from resonators with various frequency modes.

The upper limit of the detectable frequency range for the probe, denoted as the characteristic frequency (*f*_c_), is theoretically determined by the product of *I*_c_ and *R*_n_ in Josephson junctions. By combining the AC Josephson voltage–frequency parameter with the measured *I*_c_*R*_n_ value of the probes, *f*_c_ can be derived by using *f*_c_ = *I*_c_*R*_n_ × (2e/h). We obtain *I*–*V* characteristics from different batches of probes and calculate the *f*_c_ values (Fig. S6). The *f*_c_ of probes is determined by the apex size of the quartz tube and the thickness of the Nb film, which can be effectively controlled from several GHz to 200 GHz. For tracking frequencies that are much lower than the *f*_c_ value of the probe, the thermal noise may destroy the synchronization between the Josephson AC supercurrents and the external microwave [38,39] so that the capability of the probe for coherent detection is lost. Thus, probes with different *f*_c_ values are recommended to be selectively employed for microwave characterization scenarios that involve varying frequency ranges.

### Spatial imaging

**Figure 4.**
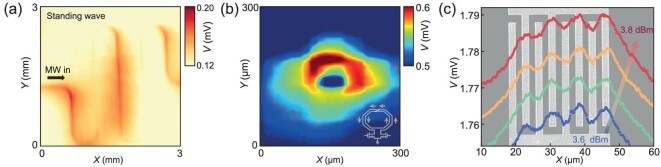
Spatial imaging by the Josephson microscope. (a) Intensity distribution of Coplanar waveguide (CPW). The signals are obtained by tracking the voltage of the probe with a bias current around *I*_c_ while scanning the CPW with an injection microwave of 12.28 GHz. The complete structural design of the CPW is shown in Fig. S7. (b) Near-field microwave above a microwave oscillator. The voltage signals of the probe are spatially tracked during the scanning process, showing a circular microwave distribution. The scanning is conducted at a height of ∼500 nm above the chip surface. The inset shows the design schematic corresponding to the imaging area. (c) 1D image of the capacitor. All curves show sawtooth microwave distributions when 3.44 GHz microwave signals with different intensities are injected. The background shows the SEM image of the capacitor, with the *y*-axis distorted for better visualization.

The coplanar waveguide (CPW) constitutes an indispensable component of the microwave resonator and has widespread application in superconducting quantum circuits [40,41]. To demonstrate the microwave imaging capabilities of Josephson microscopy, we designed a typical CPW structure composed of Nb film. The CPW is configured such that the microwave signal is fed in from the left side, while the other side remains open to create an impedance mismatch (Fig. S7). As a result, a standing wave along the central conductor always occurs, with its periodic length depending on the injection microwave frequencies. We imaged the spatial distribution of these standing waves within the CPW at a height of 10 μm above the sample surface. The Josephson probe is biased around its *I*_c_ and voltages were tracked and spatially plotted into a colormap (Fig. 4a). The result reveals a clear periodic distribution along the central conductor, with at least two cycles inside. To validate our results, we conducted a simulation that exhibits patterns that are similar to those observed in the experimental data (Fig. S8) and the wavelength of the standing wave is calculated to be ∼10.44 mm. The tip-sample distance is also crucial when detecting near-field microwave signals. Although the imaging height is much smaller than the microwave wavelength (tens of millimeters), the signals of the microwave distribution become blurred as the tip-sample distance gradually increases from 1 to 100 μm (Fig. S9), indicating the necessity for accurate microwave characterization in near-field imaging.

In addition to examining the superconducting device, we conducted near-field microwave characterization on a semiconductor voltage-controlled oscillator (VCO). Detailed descriptions can be found in the Supplementary Data (Fig. S10). Given that the chip operates within the microwave frequency band, emitted electromagnetic waves from the chip surface could be detected and analysed. The resulting image exhibits a circular pattern (Fig. 4b). Localized signal enhancement has been observed at the upper part of the circular ring, which may be attributed to the intersecting double-loop inductors at this position. Conversely, the weaker microwave signal at the lower part corresponds to the capacitance structure. The imaging characteristics are completely consistent with its circular inductance-capacitance (LC) resonant design. The finding underscores the potential of Josephson microscopy to solve electromagnetic compatibility issues in high-frequency integrated circuits (ICs).

To showcase the spatial resolution of the Josephson microscope, we selected a classic structure ‘capacitor’, which has wide applications in microwave quantum technologies [42,43]. We designed a micro-sized interdigital capacitor (Fig. S11), with a width of 2 μm for central electrodes and 2 μm gaps in between. An enlarged SEM image of the interdigital capacitor is presented in the background of Fig. 4c. Microwave signals are fed from the top electrode, while the bottom electrode is open. The characteristics of the microwave distribution are consistent with both the shape and number of interdigital electrodes. The colored curves represent the results of line scanning along the *x*-axis under different intensities of microwave injection. As the microwave intensity increases, the voltage signals detected from the probe progressively rise, allowing clear observation of the microwave signals that are emitted from the electromagnetic coupling between the interdigital electrodes. By analysing the peak-to-trough feature size and the signal-to-noise ratio, we estimate the best-effort spatial resolution to be better than 1 μm. Considering the wavelength (λ) of the feed-in microwave and a resolution of 1 μm, the Josephson microscope has reached a detecting scale of 1 × 10^–5^ λ so far. By further incorporating the tip-sample-distance control methods that are commonly employed in traditional scanning probe microscopy, such as the microscopes based on tuning forks [30] and optical interferometers [44], a nanoscale spatial resolution could be feasibly attained.

## DISCUSSION AND CONCLUSION

In summary, our study introduces low-temperature passive near-field microwave microscopy by placing nanosized Josephson mixers onto a probe. We demonstrate its remarkable capabilities in terms of a broad bandwidth of ≤200 GHz and multiple operating modes, paving the way for near-field characterization of weak signals from chip-based microwave devices. The experiments at various temperatures reveal that lower temperatures can enhance the performance of the Josephson probe (Fig. S12), making it compatible with the environment at ultra-low temperatures that are required for quantum circuits. Compared with existing techniques, the Josephson probe offers an effective methodology in terms of both spatial resolution and detecting bandwidth (Table S1), enabling the passive near-field identification of subtle high-frequency signals.

Applications of the Josephson microscope into even higher frequency ranges would be possible by optimizing the probe with different structures and/or materials, such as the fabrication of superconductor–insulator–superconductor junctions [45]. On the other hand, the Josephson probe can be used as a nanoscale bolometer, enabling non-coherent detection in the terahertz domain [46]. Our microscope offers an *in situ* and non-destructive characterization tool that is compatible with quantum technologies that operate at ultra-low temperatures. It can also directly advance the on-chip circuit design and facilitate the discovery of novel phenomena in terahertz physics [47–49], spintronics [50], metamaterials [51] and high-frequency ICs [2].

## METHODS

The fabrication procedures of the Josephson probes, set-up of the Josephson microwave microscope and parameters of the coplanar waveguide, VCO chip and interdigital capacitor are shown in the Supplementary Data.

## Supplementary Material

nwae308_Supplemental_File
