# Supplementary material for: Ultra-broadband near-field Josephson microwave microscopy
Source: Natl Sci Rev. 2024 Sep 3;12(2):nwae308. doi: 10.1093/nsr/nwae308 (PMC11827593; doi:10.1093/nsr/nwae308)
Supplement: nwae308_Supplemental_File [file nwae308_supplemental_file.docx]

*Supplementary Material*

**Ultra-broadband near-field Josephson microwave microscopy**

Ping Zhang^1,+^, Yang-Yang Lyu^1,+,*^, Jingjing Lv^1^, Zihan Wei^1,2^, Shixian Chen^1^, Chenguang Wang^1,2^, Hongmei Du^1^, Dingding Li^1^, Zixi Wang^1^, Shoucheng Hou^1^, Runfeng Su^1^, Hancong Sun^2^, Yuan Du^1^, Li Du^1^, Liming Gao^3^, Yong-Lei Wang^1,2,*^, Huabing Wang^1,2,*^, Peiheng Wu^1,2^

^1^*School of Electronic Science and Engineering, Nanjing University, Nanjing 210023, China*

^2^*Purple Mountain Laboratories, Nanjing 211111, China*

^3^*Institute of Electronic Materials and Technology, School of Materials Science and Engineering, Shanghai Jiao Tong University, Shanghai, 200240, China*

1. **Probe fabrication.**

The fabrication procedure involves a quartz tube (Beijing Zhong Cheng Quartz Glass Corporation) with a diameter of 1.2 mm. This tube is characterized by four deep grooves, each measuring 0.1 mm in width and 0.25 mm in depth, arranged at 90-degree intervals along the sidewall. Laser heating during the pulling process caused the tube to fracture into two (Sutter Instrument P-2000), resulting in a nano-tip with broken nodes reaching an approximate diameter of 100 nm. The deep grooves around the tip remained intact. Subsequently, Nb films were deposited in a specific sequence using DC magnetron sputtering (AdNaNoTek Corp.). The first deposition was executed with the probe’s sidewall facing the direction of the plasma, while the tip was tilted upwards at 30 degrees. The second deposition required a 180-degree axial rotation of the probe. For the third deposition, the apex of the probe was directed towards the plasma. This process led to the formation of Josephson junctions as weak links at the probe’s apex. During the magnetron sputtering process, the probe is maintained at room temperature, and the deposition rate is approximately 0.4 nm/s. The thickness of the bottom Nb film is consistently set at 15 nm. The thickness of Nb films on side electrodes, which significantly influence the characteristic frequencies of probes, varies between 20 nm and 40 nm across different production batches.

1. **Microscope setup.**

All transport characteristics were measured in a helium-free cryostat with low vibration (Montana S100). The probe is capsuled in a homemade shielding holder with low-pass filters and mounted on the cold head of the cryostat. The scanning capability of the microscope is provided by three-axis positioners (Attocube ANPx51 and ANPz51). DC signals from the probe were obtained using a traditional four-terminal configuration. A custom-made current source and low-noise voltage amplifier were employed to conduct transport measurements, using a 12 V lead-acid battery to minimize power supply noise. The custom-made current source is voltage-controlled and outputs a constant current from 0.1 μA to 10 mA. The custom-made low-noise voltage amplifier filters high-frequency noises and amplifies voltage signals differentially, with a maximum amplification factor of 10000 times. Both devices were controlled and read by a data acquisition card (NI PCI-6221).

All microwave signals (*f*_LO_ / *f*_IF_ / *f*_RF_) were transferred by RF cables (WITC WTF6-138-138-L & Insulated Wire Inc. KPS-1501-1.0M-KPS) and DC blocking capacitors (2 pF). There is no impedance matching between the probe and RF cables due to the substantial dynamic range of the microwave source. The *f*_LO_ signal was produced by a commercial microwave source (Agilent N5183A) and then transmitted via RF cables and a DC-blocking capacitor. A gold wire with a diameter of 50 μm, secured with silver pastes, was used to establish the connection between the capacitor and the probe. The *f*_IF_ signal resulting from frequency mixing was passed through a custom-made intermediate amplifier with an operating frequency of 0.1~1 GHz and a gain of 60 dB at room temperature, and then sent to a spectrum analyzer (Agilent N9010A). The high-frequency microwave signals were provided by a signal generator extension module (Virginia Diodes, Inc. 80-250 GHz) or a Gunn oscillator (Epsilon Lambda Electronic Corp. ELMI94/U).

The specific procedure to ascertain the tip-sample distance involves setting the probe to a fixed current and gradually moving it towards the DUT. Given the presence of Josephson junctions on the tip apex, any minimal contact between the probe and DUT could induce the influence of probes’ transport properties. When the voltage signals from the probe display fluctuations that exceed the noise level, we deduce that the probe has made contact with the DUT. To prevent any potential collision, the probe is then raised by a certain distance, which is determined by the *z*-axis value readout from the Attocube.

1. **Devices under test.**

The coplanar waveguide and interdigital capacitor devices were fabricated using a 50-nm Nb film obtained by DC magnetron sputtering onto a 0.5-mm-thick silicon substrate. The fabrication process involved photolithography and lift-off procedures to define the desired patterns. The central electrodes of the coplanar waveguide had a width of 30 μm, and the equivalent impedance was designed to be 50 Ω. The micro-sized interdigital capacitor had a width of 2 μm for central electrodes with 2 μm gaps in between.

The voltage-controlled oscillator (VCO) chip is fabricated using TSMC 28 nm technology. It is encapsulated in a passivation insulation layer with a cumulative thickness of 5.48 μm and a geometric average dielectric constant of 4.61. The oscillator operates using an inductance-capacitance (LC) resonant mode. Specifically, the inductor within the LC tank is structured as a symmetrical octagonal ring with two turns, with an outer diameter of 80 μm and a linewidth of 4.5 μm. The invisible capacitor is located below the gap of the inductor ring, employing a 2-bit switchable plug-in capacitor structure. The inductance value is approximately 520 pH, while the capacitance value is around 90 fF. The targeted oscillation frequency of VCO is roughly 22.5 GHz at room temperature. The chip operates normally at a bath temperature of around 10 K during the characterization.

**Table 1** Performance of different microwave imaging techniques

| **Type** | **Spatial**  **Resolution** | **Frequency**  **Bandwidth** | **Intensity**  **Sensitivity** | **Required**  **Conditions** |
| --- | --- | --- | --- | --- |
| Scanning near-field microwave microscopy | ~ 100 nm^[1]^ | < 60 GHz^[2]^ | Relatively low | None |
| Nitrogen-vacancy center microscopy | ~ 50 nm^[3]^ | < 120 GHz  @4.2 T^[4]^ | 130 nTHz^-1/2 [5]^ | Magnetic fields  & Laser |
| Atomic vapor cell microscopy | ~ 20 μm^[6]^ | < 500 GHz^[7]^ | 55 nVcm^-1^Hz^-1/2 [8]^ | Rydberg atoms  & Laser |
| This work | ~ 1 μm | < 200 GHz | -72 dBmHz^-1/2^ | Low temperature |

**Ref:**

1. Zheng, L., *et al*. Visualization of surface-acoustic-wave potential by transmission-mode microwave impedance microscopy. *Phys Rev Appl* 2018; **9**: 061002.
2. Kim, M., *et al*. Nondestructive high spatial resolution imaging with a 60 GHz near-field scanning millimeter-wave microscope. *Rev Sci Instrum* 2004; **75**: 684–688.
3. Chang, K., *et al*. Nanoscale imaging of current density with a single-spin magnetometer. *Nano Lett* 2017; **17**: 2367-2373.
4. Stepanov, V., *et al*. High-frequency and high-field optically detected magnetic resonance of nitrogen-vacancy centers in diamond. *Appl Phys Lett* 2015; **106**: 063111.
5. Horsley, A., *et al.* Microwave Device Characterization Using a Widefield Diamond Microscope. *Phys Rev Applied* 2018; **10**: 044039.
6. Wade, C.G., *et al.* Real-time near-field terahertz imaging with atomic optical fluorescence. *Nat Photonics* 2017; **11**: 40-43.
7. Sedlacek, J. A., *et al.* Atom-Based Vector Microwave Electrometry Using Rubidium Rydberg Atoms in a Vapor Cell, *Phys Rev Lett* 2013; **111**: 063001.
8. Ji, M., *et al.* Atomic superheterodyne receiver based on microwave-dressed Rydberg spectroscopy. *Nat Physics* 2020; **16**: 911–915.


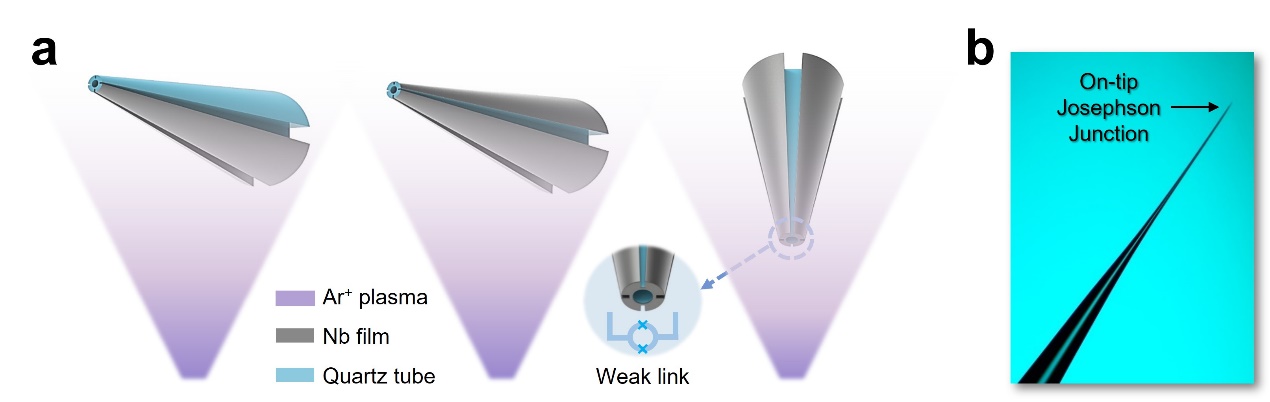


**Supplementary Figure 1.** Demonstration of Josephson probes. (a) Fabrication procedure. Niobium film (grey) was deposited onto the quartz tube (blue) by DC magnetron sputtering following three rounds: firstly, the probe is placed perpendicular to the plasma direction; secondly, it's axially rotated 180 degrees; thirdly, the probe is rotated parallel to the plasma direction, leading to the formation of weak-link Josephson junctions at the apex. (b) Optical photo. Josephson junctions are located on the apex of the probe.


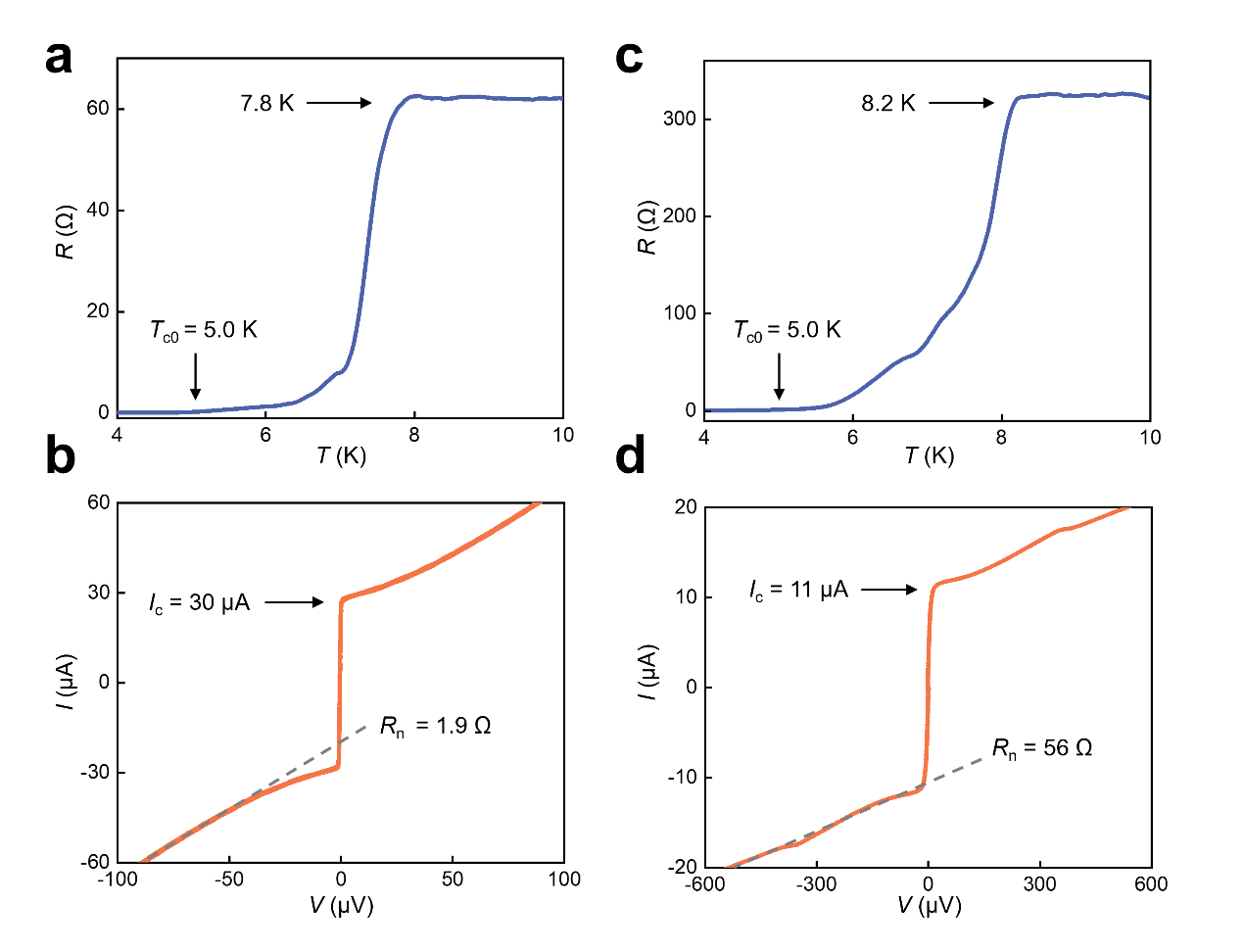


**Supplementary Figure 2.** Transport properties of Josephson probes. (a) Temperature dependence of resistance for probe #1. The resistance starts to drop around 7.8 K and reaches zero at 5.0 K. (b) *I*~*V* characteristics for probe #1. The current was sweeping in a full loop and the curve shows no hysteresis. The probe has a critical current (*I*_c_) of 30 μA and a normal resistance (*R*_n_) of 1.9 Ω. As shown in the graph, *I*_c_ is generally defined by a threshold of 5 μV, above the voltage noise level. *R*_n_ is defined by the slope of the linear region in the *I*~*V* characteristic, where the Josephson junction loses its superconducting state. (c) Temperature dependence of resistance for probe #2. The resistance starts to drop around 8.2 K and reaches zero around 5.0 K. A residual resistance of 8.9 Ω is subtracted. (d) *I*~*V* characteristics for probe #2. The probe has a critical current of 11 μA and a normal resistance of 56 Ω.


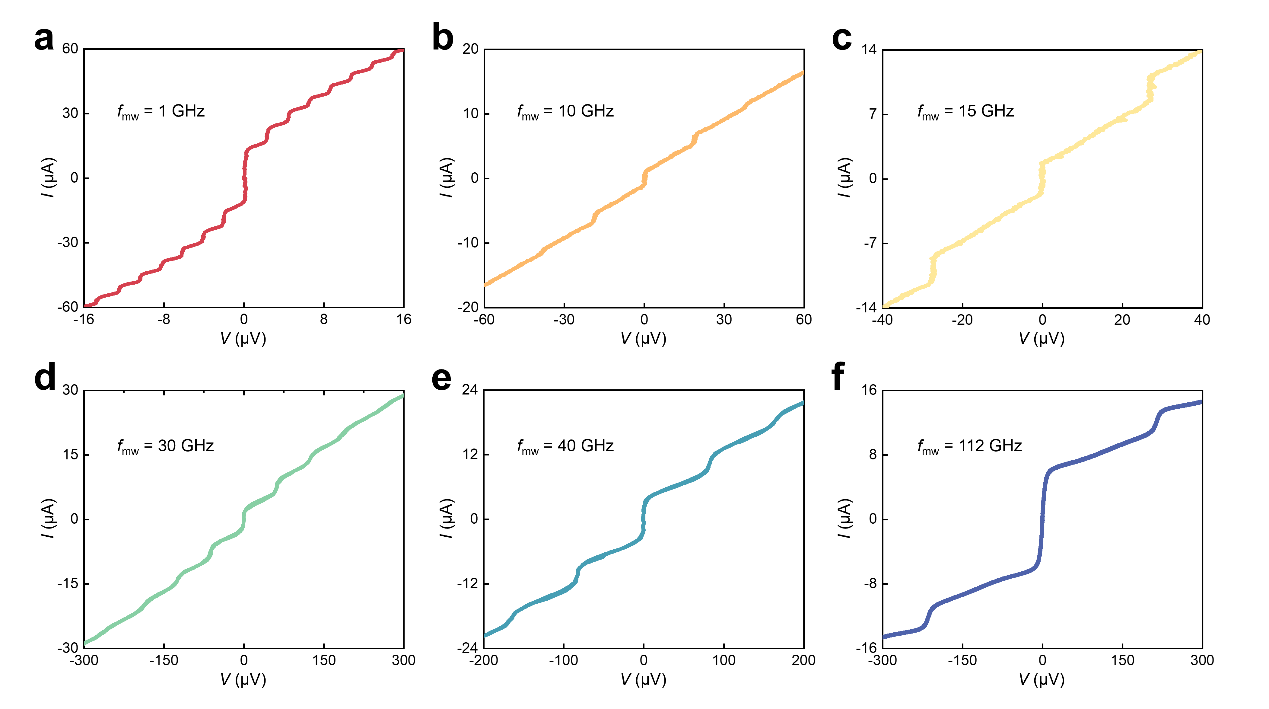


**Supplementary Figure 3.** Microwave responses of different batches of probes. Microwave signals of 1~40 GHz are provided by a commercial microwave source, while the 112 GHz microwave signal is provided by a Gunn oscillator. The presence of Shapiro steps confirms the existence of Josephson junctions on those tips, showing a repeatable procedure to fabricate Josephson probes. The rounding features of Shapiro steps may result from excess currents, ambient noises, thermal noises or the overheating effect.


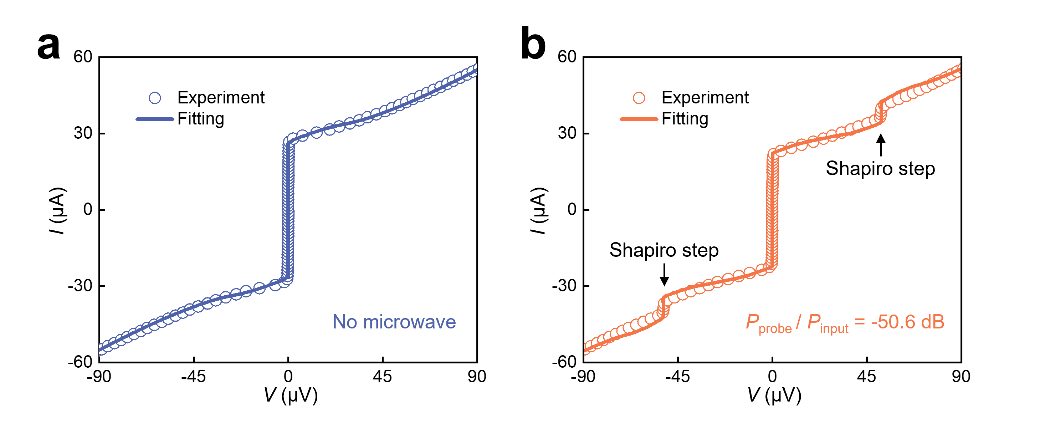


**Supplementary Figure 4.** Comparison between experiments and fitting for probe #1. (a) Fitting of original *I*~*V* characteristic. Dots, extracted from Fig. 2c, represent the experimental *I*~*V* characteristic without microwave, while solid lines are simulating results fitted by the RCSJ model. In a normalized form, the RCSJ model can be written as *i*_b_=*β*_c_d^2^*φ*/d*τ*^2^+d*φ*/d*τ*+sin*φ*. The bias current (*i*_b_) consists of *i*_dc_+*i*_ac_sin(*ωτ*)+*i*_n_sin(*ω*_n_*τ*), corresponding to DC bias currents, a.c. currents and currents produced by external noise, respectively. In the formula, *ω* is the Josephson plasma period, *ω*_n_ is the external noise period, *φ* is the Josephson phase difference and *β*_c_ is the Stewart-McCumber parameter. (b) Fitting of *I*~*V* characteristic under microwave injection. Dots, extracted from Fig. 2a, represent the experimental *I*~*V* characteristic under a 25 GHz microwave signal with an intensity (*P*_input_) of -10 dBm. The fitting curve (solid line) shows the same feature as that in the experimental data, indicating that the microwave intensity theoretically acting on the probe (*P*_probe_) is around -60.6 dBm. Thus, we determined the attenuation coefficient of the system (*P*_probe_/*P*_input_) to be -50.6 dB, resulting from the impedance mismatching.


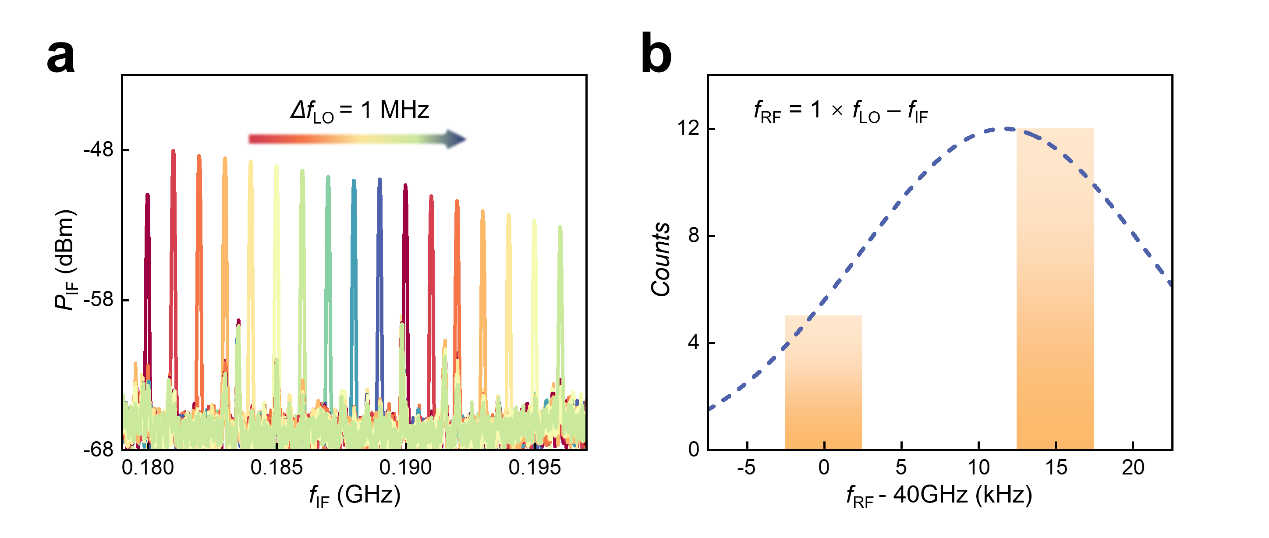


**Supplementary Figure 5.** Fundamental frequency mixing for probe #1. (a) Spectra of resulting *f*_IF_. Peaks of the spectra indicate the variations of *f*_IF_ output, while *f*_LO_ is changing from 40.180 GHz to 40.196 GHz, with an interval of 1 MHz and 17 times in total (from left to right). (b) Calculated *f*_RF_ values. By fitting the equation of *f*_RF_ = 1×*f*_LO_ - *f*_IF_ from the spectrum in (**a**), the frequency of *f*_RF_ could be reconstructed. Due to the accuracy limitation of spectral data, 17 calculated *f*_RF_s from **(a)** are discretely distributed around 40 GHz. The histograms (orange) and distributions (blue) indicate the counts and fitting frequency of *f*_RF_ signals. The center frequency of *f*_RF_ is determined to be 40 GHz+13.9 kHz. The *f*_RF_ values in the *x*-axis are subtracted by a value of 40 GHz.


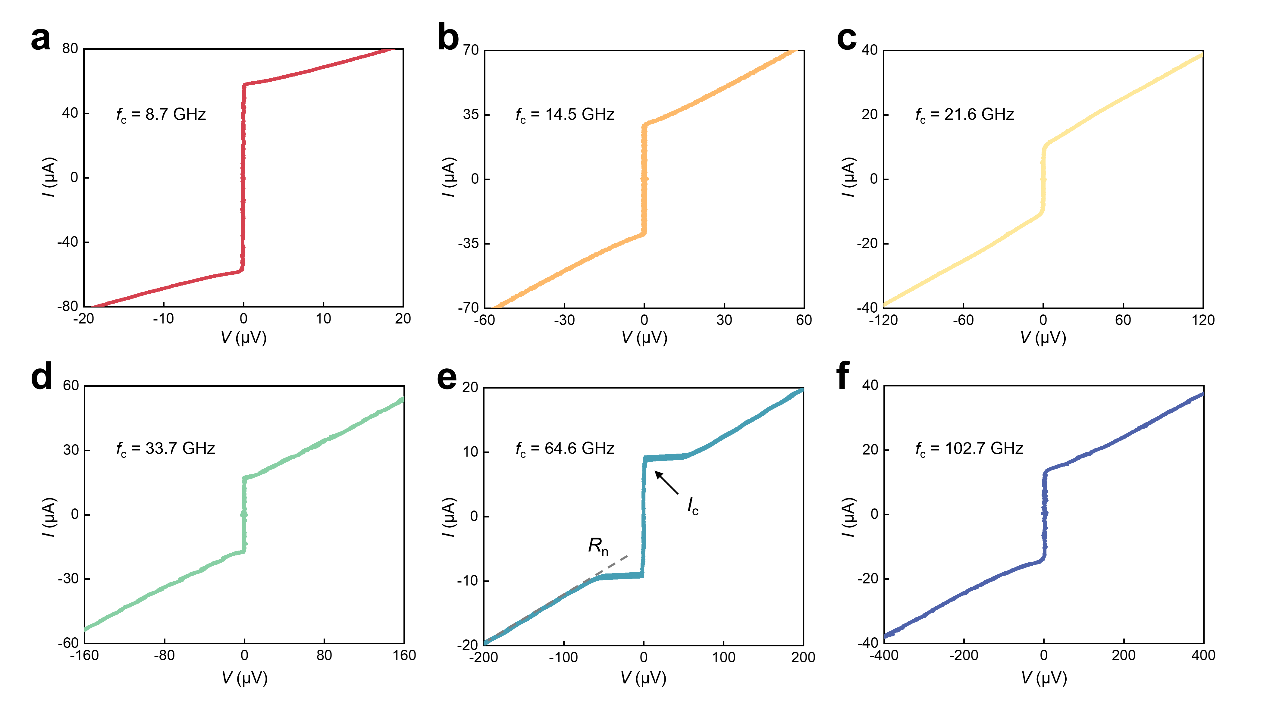


**Supplementary Figure 6.** Transport properties of different probes. The bias current is sweeping in a full loop and *I*~*V* characteristics normally show no hysteresis. The critical currents (*I*_c_) of probes typically range from 1 μA to 100 μA. The upper limit of frequency detection (*f*_c_) shown on the left-top corner can be calculated by *f*_c_=*I*_c_*R*_n_×(2e/h), indicating the frequency range where these probes can conduct coherent detection. As shown in (e), *I*_c_ is generally defined by a threshold of 5 μV, above the noise level. The normal resistance (*R*_n_) is defined by the slope of the linear region in the *I*~*V* characteristic, where the Josephson junction loses its superconducting state. Due to certain reasons like excess currents, the defined *R*_n_ is slightly higher than its intrinsic *R*_n_, but it is sufficient to estimate the value of *f*_c_.


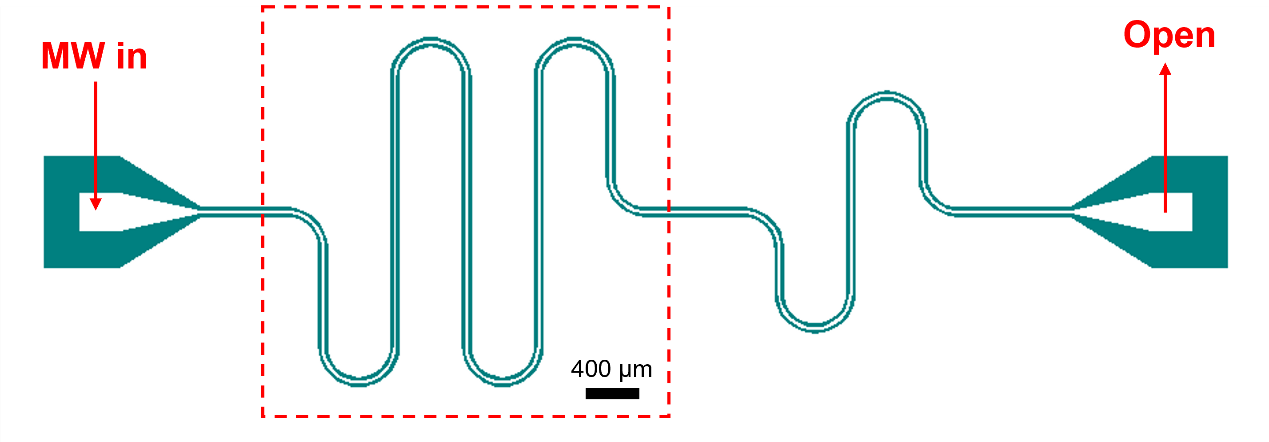


**Supplementary Figure 7.** Design of the coplanar waveguide (CPW). The central conductor of the CPW has a width of 30 μm and an equivalent impedance of 50 ohms. The microwave signal is fed in from the left side, while the right side remains open-circuited. The red frame marks out the scanning region, which reaches the maximum area (3×3 mm^2^) offered by the scanning platforms.


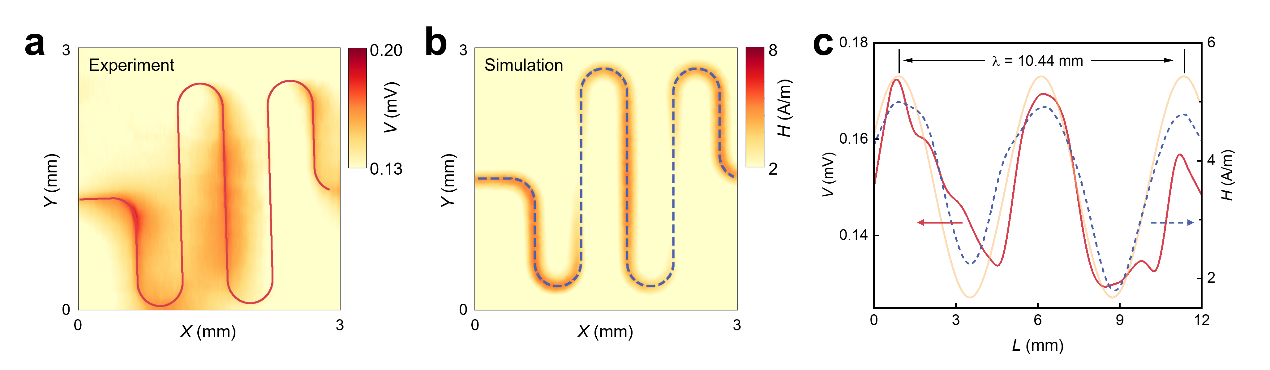


**Supplementary Figure 8.** Analysis of the standing wave. (a) Experimental imaging. The data is derived from Fig. 4a, with the red solid line indicating the position of the central conductor in the CPW. (b) Simulated imaging. The simulation was conducted using COMSOL, with the model size precisely matching that of CPW. The blue dashed line indicates the position of the central conductor in the CPW. (c) Fitting of standing wave. The red curve illustrates the spatial distribution of the probe's voltages along the line in **(a)**. The blue dashed line represents the fitting result from **(b)**, yielding a wavelength of approximately 10.44 mm. The orange line corresponds to a standard sinusoidal curve for comparison.


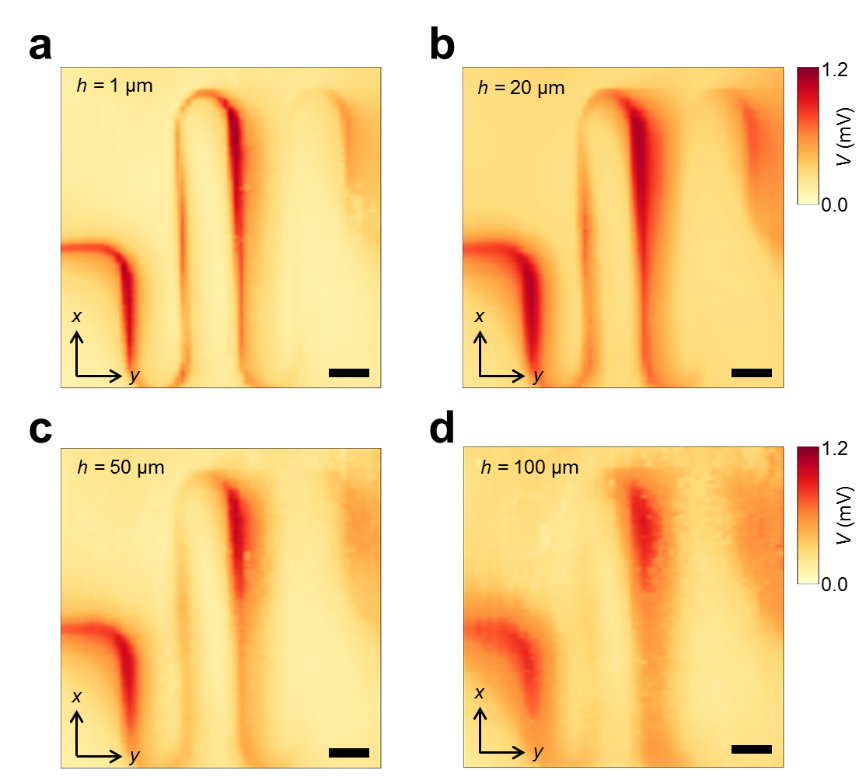


**Supplementary Figure 9.** Intensity distribution of CPW on different heights. The signals are obtained by tracking the probe’s voltages while scanning the CPW. The injecting microwave is at 2.90 GHz. The tip-sample distances (*h*) vary from 1 μm to 100 μm in (a)-(d). All color bars are shown in the same range for comparison. Scale bar, 400 μm.


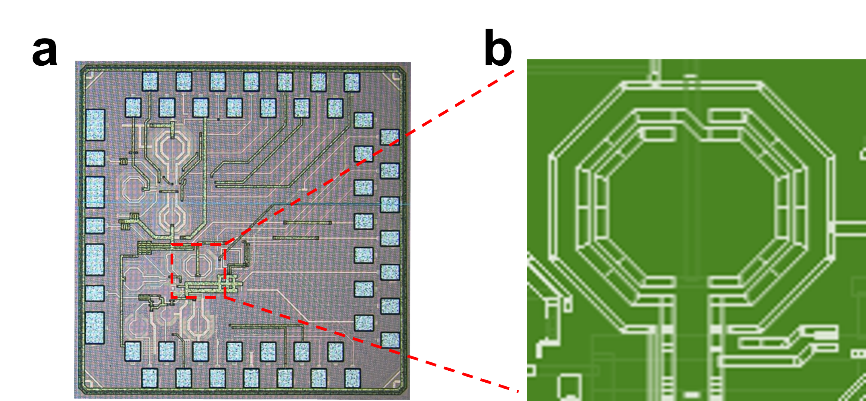


**Supplementary Figure 10.** Images of the semiconductor chip. (a) Optical photo of the chip. The red dashed box marks out the imaging area in Fig. 4b and Fig. S10b. (b) Circuit diagram of the VCO. The main structure in the figure is a symmetrical octagonal inductor, forming an LC resonant cavity together with an invisible capacitor underneath.


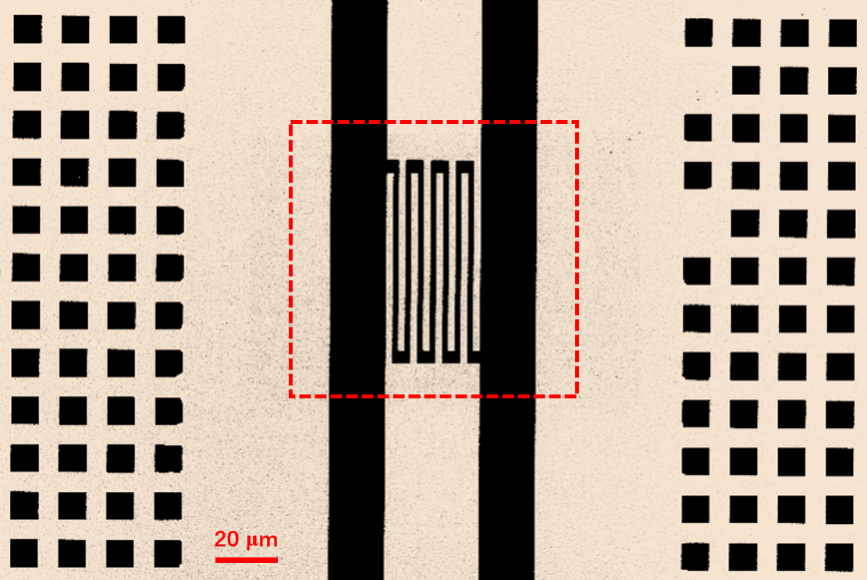


**Supplementary Figure 11.** SEM image of the interdigital capacitor. The areas of Nb film are marked out in orange by false color, while black regions correspond to the silicon substrate. The red dashed box highlights the position of the interdigital capacitor.


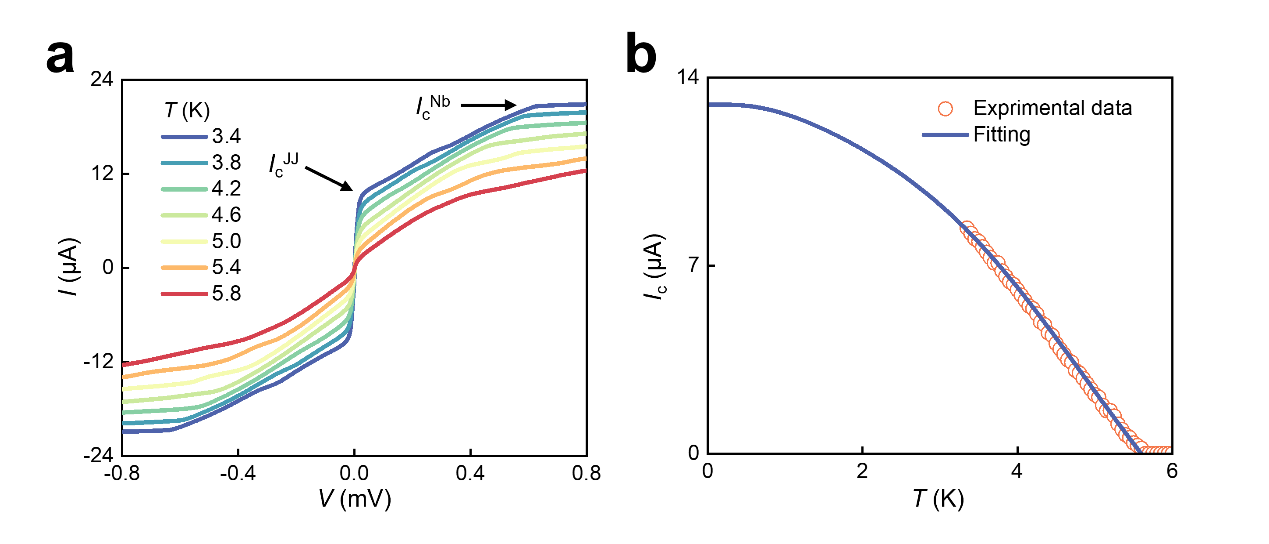


**Supplementary Figure 12.** Temperature dependence of the probe. (a) *I*~*V* characteristics at different temperatures. The critical currents of the Josephson junction (*I*_c_^JJ^) and Nb film (*I*_c_^Nb^) are marked out in the graph. (b) Temperature dependence of the probe’s *I*_c_^JJ^. Red dots represent the probe’s *I*_c_s extracted from (**a**), by defining the probe’s *I*_c_ at a differential resistance of 5 Ω. The blue dashed line indicates the fitting curve based on the Ambegaokar-Baratoff formula $J_{c}(T)=\frac{\pi\Delta(T)}{2eR_{n}}tanh\frac{\Delta(T)}{2k_{B}T}$, where *Δ*(T) is the superconducting energy gap, *R*_n_ is the normal resistance of the junction, *e* is the electron charge and *k*_B_ is the Boltzmann constant. The temperature dependence experiment reveals that the *I*_c_ of the junction increases with decreasing temperature, reaching the maximum at millikelvin. Since *I*_c_*R*_n_ defines the probe's detecting responsivity and upper limit of detecting frequency, the increased *I*_c_ effectively raises the probe’s performance at ultra-low temperatures.
